# Olive Mill Wastewater Polyphenol-Enriched Fractions by Integrated Membrane Process: A Promising Source of Antioxidant, Hypolipidemic and Hypoglycaemic Compounds

**DOI:** 10.3390/antiox9070602

**Published:** 2020-07-10

**Authors:** Rosa Tundis, Carmela Conidi, Monica R. Loizzo, Vincenzo Sicari, Alfredo Cassano

**Affiliations:** 1 Department of Pharmacy, Health and Nutritional Sciences, University of Calabria, 87036 Rende (CS), Italy; rosa.tundis@unical.it (R.T.); monica_rosa.loizzo@unical.it (M.R.L.); 2 Institute on Membrane Technology, ITM-CNR, 87036 Rende (CS), Italy; c.conidi@itm.cnr.it; 3 Department of Agricultural Science, Mediterranean University of Reggio Calabria, 89123 Reggio Calabria, Italy; vincenzo.sicari@unirc.it

**Keywords:** olive mill wastewater, integrated membrane processes, ultra-high-performance liquid chromatography (UHPLC), polyphenols, antioxidants, obesity

## Abstract

The valorisation of food wastes is a challenging opportunity for the green, sustainable, and competitive development of industry. The recovery of phenols contributes to the sustainability of olive waste sector, reducing its environmental impact and promoting the development of innovative formulations of interest for pharmaceutical, nutraceutical, and cosmeceutical applications. In this work, olive mill wastewater was treated through a combination of microfiltration (MF), nanofiltration (NF), and reverse osmosis (RO) in a sequential design to produce polyphenol-enriched fractions that have been investigated for their chemical profile using ultra-high-performance liquid chromatography (UHPLC), and their potential antioxidant, hypolipidemic, and hypoglycaemic activities. RO retentate exhibited the highest content of hydroxytyrosol, tyrosol, oleuropein, verbascoside, vanillic acid, and luteolin. In particular, a content of hydroxytyrosol of 1522.2 mg/L, about five times higher than the MF feed, was found. RO retentate was the most active extract in all in vitro tests. Interestingly, this fraction showed a 2,2′-azino-bis(3-ethylbenzothiazoline-6-sulfonic) acid (ABTS) radicals scavenging activity with an IC_50_ value of 6.9 μg/mL and a potential inhibition of lipid peroxidation evaluated by the β-carotene bleaching test with IC_50_ values of 25.1 μg/mL after 30 min of incubation. Moreover, RO retentate inhibited α-amylase and α-glucosidase with IC_50_ values of 65.3 and 66.2 μg/mL, respectively.

## 1. Introduction

The olive oil production is a strategic economic activity within the agro-industrial production of Mediterranean countries. However, the growing interest in olive oil consumption has spread the production to non-traditional producing countries such as Australia, South America and New Zealand. The continuous three-phase decanting system for olive pressing involves the addition of water (up to 50 L/100 kg olive paste) with a production of large amounts of olive mill wastewaters (OMWs). It has been estimated that the water consumption to produce 1 ton of olive oil can reach up to 14.5 million L [1], while the annual world production of OMWs comes from 10 to over 30 million m^3^ [2]. OMWs are dark-brown liquids with an acid reaction (pH between 3 and 6) and are considered as one of the most polluting effluents of the agro-food industries due to their high polluting load. Their composition is strongly influenced by the variety of olives and seasonality. Typically, they contain water (83–94%), organic substances (4–18%), including carbohydrates, pectins, mucilage, tannins, organic acids, phenolic compounds, lipids and inorganic substances.

In general, the high phenolic content makes olive mill wastewater difficult to biodegrade, creating severe environmental and economic problems for its disposal.

Several treatment procedures, including physical, chemical, biological or combined technologies have been evaluated to reduce the organic load and toxicity of OMWs, including aerobic and anaerobic digestion, flocculation, sedimentation, evaporation, electrocoagulation, advanced oxidation processes [3,4,5]. Most of these methods, such as thermal processes and advanced oxidation methods, are very effective but they are limited by high operating costs. The combination of physico-chemical and biological processes is not completely successful, since it requires a longer lag phase before the biological treatment. As a consequence, affordable and sustainable solutions being able to meet quality required by environmental standards have not been found so far. On the other hand, phenolic compounds are well known for their healthy beneficial properties, due to their antioxidant, anti-inflammatory, anticancer, cardio-protective, and hypoglycaemic properties [6,7,8] and are widely recognized as valuable molecules in several fields, particularly pharmaceutical and nutraceutical [9]. Cosmetic formulations are also of interest as alternatives to several synthetic ingredients to treat specific skin diseases, such as allergic and irritant contact dermatitis, phototoxic reactions, and photo-allergic reactions [10].

As reported by several studies, oxidative stress is involved in the pathogenesis and/or progression of some diseases, including obesity [11,12]. Obesity increases the risks for various diseases, such as hypertension, cardiovascular diseases, type II diabetes, and others. Moreover, it decreases the functional capacity and the quality of life, increasing morbidity and mortality. Therefore, preventing and/or treating obesity would be beneficial. One of the crucial steps in the treatment of obesity is the reduction in calorie intake. However, several studies have proved a key role of natural polyphenols as multifunctional anti-obesity agents. Among them, epigallocatechin gallate, resveratrol, catechin, quercetin, procyanidins and anthocyanins are those that are generating more interest for their anti-obesity properties [13]. Phenols act as anti-obesity agents through different mechanisms, including the inhibition of enzymes such as pancreatic lipase. This enzyme is responsible for the hydrolysis of about 50–70% of consumed lipids [14]. Standardised static in vitro digestion methods suitable to determine the bioaccessibility or the digestibility of these compounds have also been proposed as an alternative to human trials [15].

Several techniques can be used individually or in a combined form to recover phenolic compounds from OMWs. They include solvent extraction [16], ultrasound treatment [17], liquid-liquid solvent extraction [18], cloud point extraction [19], adsorption and ion exchange [20]. Unfortunately, almost all tested processes suffer from serious inconveniences, such as high cost and low efficiency. In this regard, membrane separation processes have gained great attention in the last few years as alternative or complementary technologies to the existing ones thanks to their specific characteristics, which include small area-requirement, low energy-consumption, no additives, mild operating conditions, high separation efficiency and easy scaling up [21,22]. In particular, microfiltration (MF), ultrafiltration (UF), nanofiltration (NF) and reverse osmosis (RO) successfully meet the requirement for the recovery, purification and concentration of bio-phenols from OMWs with regard to their specific molecular weight cut-off (MWCO). Integrated membrane processes, based on a sequential combination of these processes, have been investigated in the logic to produce specific fractions enriched in phenolic compounds and purified water to be reused for irrigation or process water [23]. In these approaches, MF and UF are primarily used as pre-treatment steps to remove undesired compounds from phenolic compounds, while NF and RO are used for fractionation and/or concentration of MF or UF permeates [24,25,26,27,28,29,30].

The integration of membrane operations or between membrane technology and conventional separation systems is specifically designed in order to achieve benefits from the synergy among the different unit operations and to overcome typical inherent limitations of single membrane units, such as membrane fouling, a high concentration of suspended solids, viscosity and osmotic pressure in relation to the target quality.

Innovative membrane operations, such as membrane distillation [31,32], forward osmosis [33], and osmotic distillation [34,35,36], have also been tested alone or in integrated processes for the concentration of phenolic compounds from OMWs.

The main goals of this work are: (a) to evaluate the performance of an integrated membrane systems based on conventional pressure-driven membrane operations, including MF, NF and RO, for the recovery, purification and concentration of phenolic compounds from OMWs; (b) to chemically characterize the obtained enriched fractions; (c) to investigate the antioxidant, hypoglycaemic, and hypo-lipidemic properties of these fractions in order to prospect and to propose a potential use in pharmaceutical, nutraceutical, and cosmeceutical industries.

## 2. Materials and Methods

### 2.1. Chemicals and Reagents

Solvents of HPLC grade were purchased from Carlo Erba Reagents (Milan, Italy), while solvents of analytical grade were obtained from VWR International s.r.l. (Milan, Italy). Acarbose was purchased from Serva (Heidelberg, Germany). Folin-Ciocalteu reagent, Tween 20, ascorbic acid, butylated hydroxytoluene (BHT), sulphuric acid, sodium carbonate, propyl gallate, 2,2-diphenyl-1-picrylhydrazyl (DPPH), tripyridyltriazine (TPTZ), 2,2′-azino-bis (3-ethylbenzothiazoline-6-sulfonic) acid (ABTS) solution, β-carotene, linoleic acid, orlistat, 4-nitrophenyl octanoate (NPC), maltose, α-amylase from porcine pancreas, α-glucosidase from *Saccharomyces cerevisiae*, *o*-dianisidine dihydrochloride, 4-nitrophenyl octanoate (NPC), porcine pancreatic lipase, peroxidase/glucose oxidase (PGO), hydroxytyrosol, tyrosol, 4-hydroxyphenyl acetate, vanillic acid, caffeic acid, *p*-coumaric acid, ferulic acid, verbascoside, luteolin, and oleuropein were purchased from Sigma-Aldrich S.p.a. (Milan, Italy).

### 2.2. Olive Mill Wastewaters (OMWs)

Raw OMWs obtained from a 3-phase centrifugation process were supplied by Olearia San Giorgio (San Giorgio Morgeto, Reggio Calabria, Italy). Their initial pH (about 5) was adjusted up to 2.6 by adding sulphuric acid (95–98% purity) in order to achieve the coagulation and precipitation of suspended solids according to Stokes law [37]. Then, OMWs were submitted to a prefiltration through a nylon filter followed by a filtration with a wire mesh filter of 30–40 μm. This process allowed us to reduce the suspended solids content of acidified wastewaters from 11.5% to 2.0%.

### 2.3. Membrane Processing: Experimental Set-Up

The pre-treated OMWs (feed MF) were microfiltered by using a pilot unit equipped with a multichannel ceramic (TiO_2_) membrane (Ceram Inside, from Tami Industries) having a nominal pore size of 0.1 μm and a membrane surface area of 0.35 m^2^. Experiments were performed in a continuous mode of operation in which the feed solution was continuously fed on the separating surface of the membrane, while permeate was continuously collected from the other side. The MF system was operated at a transmembrane pressure (TMP) of 1.6 bar, a feed flowrate of 6 m^3^/h and a temperature of 25 ± 1 °C up to a recovery factor of 94%.

**Figure 1:**
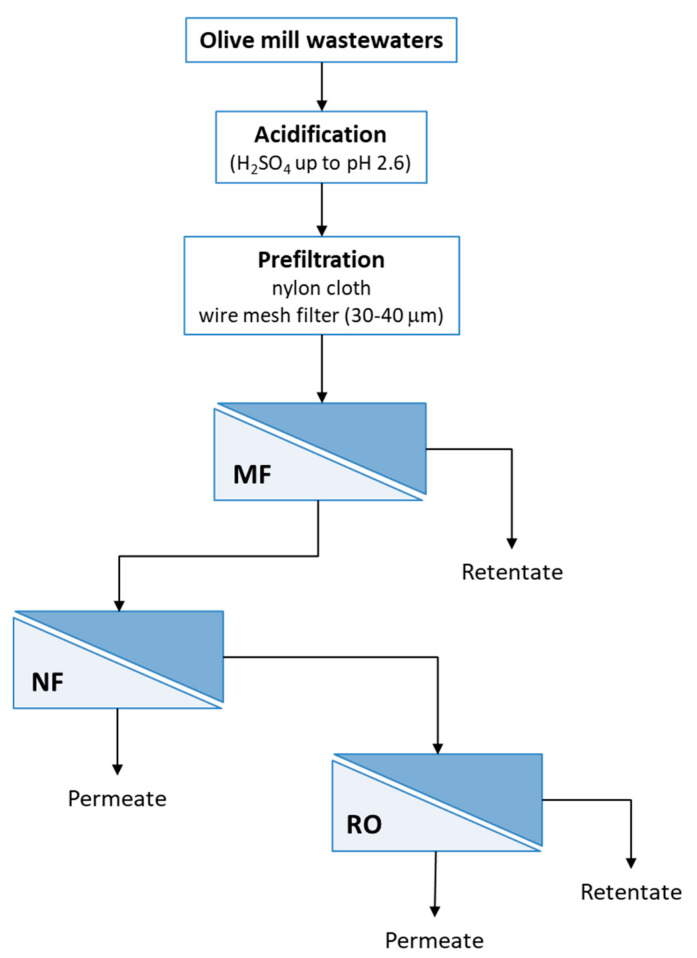
Block diagram of investigated process (MF, microfiltration; NF, nanofiltration; RO, reverse osmosis).

The microfiltered solution (MF permeate) was submitted to a NF step. It was performed by using a bench plant (Matrix Desalination Inc., Fort Lauderdale, FL, USA) equipped with a polyamide membrane module in spiral-wound configuration with a molecular weight cut-off of 150–300 Da and a membrane surface area of 2.6 m^2^ (DK from GE Osmonics, Minnetonka, MN, USA). NF experiments were performed according to a batch concentration configuration (with full recycling of retentate in the feed tank) in selected operating conditions of pressure (7 bar), axial feed flowrate (188 L/h) and temperature (25 ± 1 °C) up to a volume reduction factor (VRF) of 4. The NF permeate was concentrated by RO using the same plant equipped with a polyamide membrane module in spiral-wound configuration with a MgSO_4_ rejection of 99.4% and a membrane surface area of 2.34 m^2^ (SWC-2540 from Hydranautics/Nitto Denko, Oceanside, CA, USA). The RO system was operated according to a batch concentration operation at an operating pressure of 16 bar and a temperature of 25 ± 1 °C up to a VRF of 2. The block diagram of the process is illustrated in Figure 1.

### 2.4. Ultra-High Performance Liquid Chromatography (UHPLC) Analyses

Identification and quantification of chemical compounds were performed by using ultra-high-performance liquid chromatography (UHPLC) following the method previously described by Romeo et al. [38]. This system consisted of an UHPLC PLATIN blue (Knauer, Germany) equipped with a binary pump system using a Knauer column C18 (100 × 2 mm, 1.8 μm) coupled with a Photo Diode Array Detector (PDA)-1 PLATIN blue (Knauer, Germany). Clarity 6.2 (Clarity System Limited, Totonto, ON, Canada) was used as the software.

Samples were preventively filtered by using a syringe filter of 0.22 μm. The mobile phase consisted of a combination of A (water acidified with acetic acid, pH 3.1) and B (acetonitrile). The following gradient elution was used: (i) 0–3 min 95% A and 5% B; (ii) 3–15 min 95–60% A and 5–40% B; and (iii) 15–15.5 min 60–0% A and 40–100% B. External standards (at concentration ranged from 1 to 100 mg/L), such as caffeic acid, *p*-coumaric acid, ferulic acid, 4-hydroxyphenyl acetate, hydroxytyrosol, luteolin, oleuropein, tyrosol, vanillic acid, and verbascoside, were used for the quantification. The results are expressed as mg/L.

### 2.5. Pancreatic Lipase Inhibition Assay

Pancreatic lipase inhibitory activity was determined as previously described by using orlistat as a positive control [39]. 4-nitrophenyl octanoate (NPC), 5 mM in dimethyl sulfoxide solution, and an aqueous solution of porcine pancreatic lipase (1 mg/mL), and Tris-HCl buffer (pH 8.5) were prepared.

The mixture, consisting of sample (at concentration in the range from 2.5 to 40.0 mg/mL), enzyme, NPC, and buffer, was incubated for 30 min at 37 °C, and the absorbance was read at 405 nm.

### 2.6. α-Amylase Inhibitory Activity Test

In this assay, the α-amylase solution was prepared by dissolving 25.3 mg of the enzyme in 100 mL of cold distilled water [40]. As previously reported, the addition of 3,5-dinitrosalicylic acid solution to the sodium potassium tartrate solution allowed us to prepare the colorimetric reagent.

Samples at concentrations in the range 12.50–1000 μg/mL were added to the starch solution and left to react at room temperature with the enzyme. The absorbance was read at 540 nm. Acarbose was used as a positive control.

### 2.7. α-Glucosidase Inhibitory Activity Test

The α-glucosidase inhibitory activity test was done following the procedure previously described [40]. In brief, samples at concentrations in the range 12.50–1000 μg/mL were stirred into a maltose solution previously prepared at 37 °C. The reaction was started by adding the α-glucosidase enzyme and was stopped after 30 min of incubation at 37 °C by adding a perchloric acid solution.

The supernatant of tube of the first step was mixed with *o*-dianisidine colour reagent (DIAN) solution and peroxidase-glucose oxidase (PGO) system-colour reagent solution and was left to incubate at 37 °C for 30 min. The absorbance was measured at 540 nm.

### 2.8. Antioxidant Activity

The antioxidant activity was assessed by using the following in vitro tests: (a) β-carotene bleaching test; (b) 2,2’-azino-bis(3-ethylbenzothiazoline-6-sulphonic) acid (ABTS) diamonium salts test; (c) 2,2-diphenyl-1-picrylhydrazyl (DPPH); and (d) ferric reducing activity power (FRAP).

The β-carotene bleaching test was done following the procedure previously described [41], using propyl gallate as a positive control. A solution of linoleic acid, Tween 20, and β-carotene was prepared and added to a 96-well microplate containing samples in concentrations in the range 2.5–100 μg/mL. The microplate was located at 45 °C. The absorbance was read against a blank at (i) *t* = 0, (ii) after 30 min of incubation, and (iii) after 60 min of incubation at 470 nm.

The ABTS test was applied to investigate the radicals scavenging ability of samples by using a procedure previously described [41]. Ascorbic acid was the positive control. In brief, a solution of ABTS radical cation was mixed with a potassium persulphate solution and stored at 25 °C. After 12 h, the obtained solution was diluted with ethanol and read to obtain an absorbance of 0.70 at 734 nm. Then, this solution was added to samples at concentrations in the range 1–400 μg/mL and after 6 min, the absorbance was read at 734 nm.

In the DPPH test, a DPPH solution was added to the samples at concentrations in the range 1–1000 μg/mL and incubated at room temperature [41]. After 30 min, the absorbance was read at 517 nm. Ascorbic acid was the positive control.

In FRAP assay, samples are tested at the concentration of 2.5 mg/mL [41]. The FRAP reagent was prepared by mixing a tripyridyltriazine (TPTZ) solution, FeCl_3_, HCl, and acetate buffer and was added to the tubes containing samples. The absorbance was measured at 595 nm after 30 min of incubation at room temperature. The positive control was butylated hydroxytoluene (BHT).

### 2.9. Statistical Analysis

Data are reported as means ± standard deviation (S.D.). IC_50_ values (the concentration giving 50% inhibition) were calculated by using Prism GraphPad Prism version 4.0 for Windows (GraphPad Software, San Diego, CA, USA). Differences within and between groups were assessed by one-way analysis of variance test (ANOVA), and multi-comparison Dunnett’s test (α = 0.05). Tukey’s test was applied to determine any significant difference in chemical parameters among investigated samples.

## 3. Results

### 3.1. Membranes Productivity

**Figure 2:**
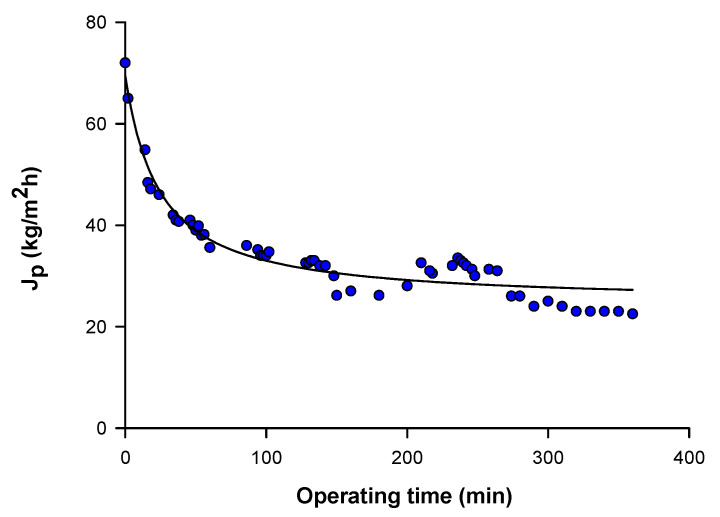
Microfiltration of pre-treated olive mill wastewaters. Time course of permeate flux (transmembrane pressure, 1.6 bar; feed flowrate, 6 m^3^/h; temperature, 25 ± 1 °C).

Figure 2 shows the time course of permeate flux in the treatment of pre-treated OMWs with the MF membrane in selected operating conditions up to a recovery factor of 94%. During the first 50 min of membrane filtration, the permeate flux declined from 72 to 39 kg/m^2^⋅h; then, it continued to decrease until reaching a steady-state value of 22 kg/m^2^⋅h. This behaviour can be attributed to typical concentration polarization and the fouling phenomenon [25,27].

**Figure 3:**
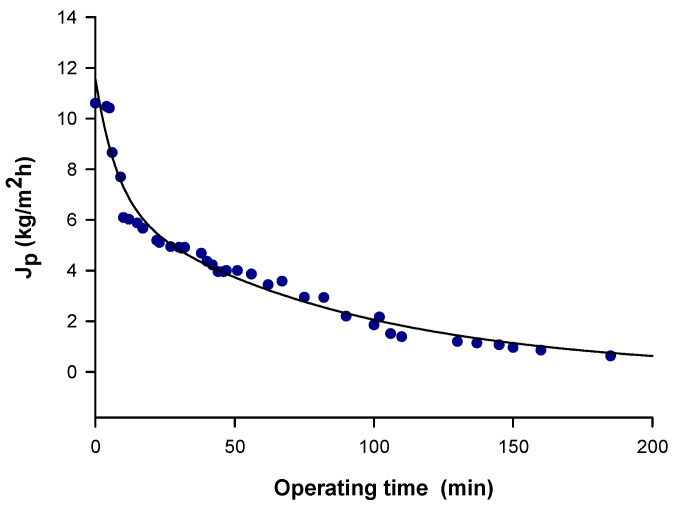
Nanofiltration of microfiltered olive mill wastewaters. Time course of permeate flux (transmembrane pressure, 7 bar; feed flowrate, 188 L/h; temperature, 25 ± 1 °C).

The NF of microfiltered OMWs in selected operating conditions produced an initial permeate flux of 10.6 kg/m^2^⋅h; the permeate flux declined rapidly in the early stage of filtration, then gradually levelled off as time progressed and eventually reached a steady-state value of about 1 kg/m^2^⋅h (Figure 3).

**Figure 4:**
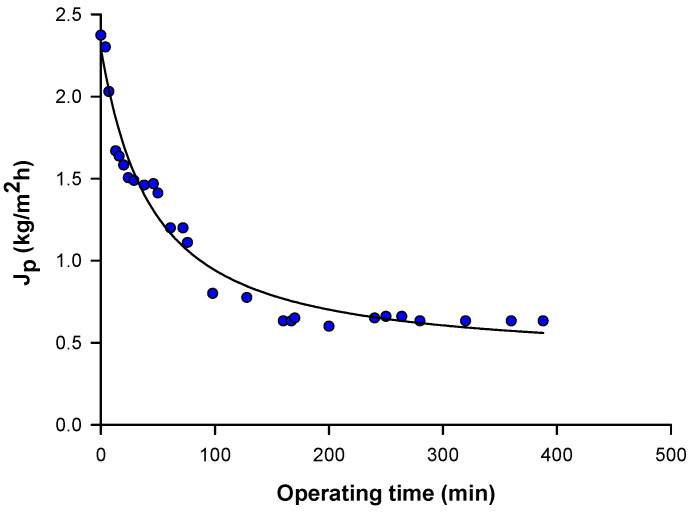
Reverse osmosis of nanofiltered olive mill wastewaters. Time course of permeate flux (transmembrane pressure, 16 bar; temperature, 25 ± 1 °C).

The NF permeate was concentrated by RO. Figure 4 shows the time course of the permeate flux in selected operating conditions: initial flux and steady-state permeate fluxes were of about 2.4 and 0.6 kg/m^2^⋅h, respectively.

### 3.2. Chemical Profile

All samples were analysed by UHPLC by using caffeic acid, *p*-coumaric acid, ferulic acid, 4-hydroxyphenyl acetate, hydroxytyrosol, luteolin, oleuropein, tyrosol, vanillic acid, and verbascoside as selected standard compounds. As reported in Table 1, the feed showed hydroxytyrosol (373.3 ± 4.8 mg/L), oleuropein (106.8 ± 4.0 mg/L), tyrosol (89.7 ± 2.1 mg/L), and 4-hydroxyphenyl acetate (72.6 ± 1.8 mg/L) as the most abundant compounds. The other compounds were quantified with values in the range of 6.4–29.4 mg/L.

As expected, RO-R is the fraction that showed the highest bioactive compounds content. In particular, the hydroxytyrosol content (1522.2 ± 7.3 mg/L) was about five times higher than the MF feed. Other identified compounds in the RO retentate were tyrosol and oleuropein with values of 519.0 ± 6.2 and 510.0 ± 5.5 mg/L, respectively, followed by verbascoside, vanillic acid, and luteolin (130.9 ± 1.2, 116.2 ± 3.1, and 82.8 ± 3.1 mg/L, respectively).

The NF-R sample showed the same trend of composition of RO-R, with hydroxytyrosol, oleuropein, tyrosol, vanillic acid, verbascoside as the dominant constituents.

In the RO permeate, only hydroxytyrosol (18.8 ± 1.2 mg/L), tyrosol (5.0 ± 0.6 mg/L), vanillic acid (1.4 ± 0.1 mg/L), and caffeic acid (0.5 ± 0.03 mg/L) were identified. The same trend was observed with the NF-P sample. However, in this sample, a higher content of hydroxytyrosol (268.3 ± 1.2 mg/L) was detected. Oleuropein and verbascoside were not detected in both NF and RO permeates.

**Figure 5:**
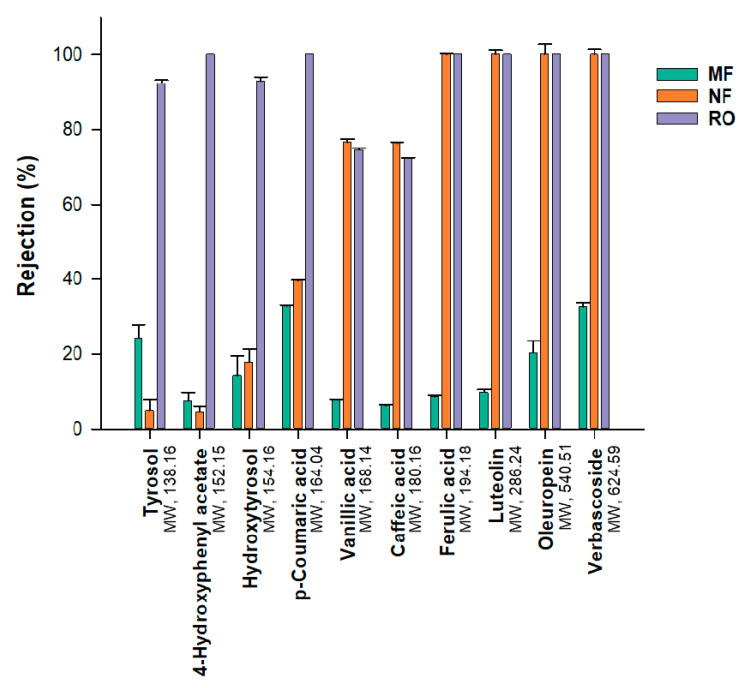
Rejection of microfiltration (MF), nanofiltration (NF) and reverse osmosis (RO) membranes towards phenolic compounds.

Figure 5 shows the rejection of selected membranes towards phenolic compounds. As expected, lower rejection values were observed for the MF membrane (in the range 7.7–32.8%); on the other hand, most parts of phenolic compounds were completely retained by the RO membrane. For the NF membrane, the rejection values measured for compounds with a molecular weight higher than 180 g/mol were similar to those observed for the RO membrane.

### 3.3. In Vitro Biological Activities

Olive mill wastewater and its polyphenol-enriched fractions obtained by MF, NF and RO have been investigated as potential agents to inhibit enzymes linked to these diseases such as α-amylase, α-glucosidase, and lipase. All samples inhibited enzymes in a concentration-dependent manner. Data (IC_50_ values) are reported in Table 2. As expected, the RO retentate is the most active sample.

In the α-amylase inhibitory activity test, the RO retentate inhibited the enzyme with an IC_50_ value of 65.3 ± 1.7 μg/mL, followed by NF retentate (IC_50_ value of 122.6 ± 2.7 μg/mL). The other samples exhibited IC_50_ values in the range 253.7–470.9 μg/mL. The same trend was observed in the α-glucosidase inhibitory test. RO retentate showed an IC_50_ value of 66.2 μg/mL, followed by NF retentate and MF permeate (IC_50_ values of 116.0 ± 2.5 and 125.4 ± 2.7 μg/mL, respectively).

Pancreatic lipase inhibitory activity has been widely used for the investigation of the potential efficacy of compounds as anti-obesity agents. RO retentate inhibited the enzyme with an IC_50_ value of 175.6 ± 2.9 μg/mL. Interesting results were also obtained by NF retentate and MF permeate (IC_50_ values of 183.1 ± 2.9 and 211.0 ± 3.4 μg/mL, respectively).

Taking into account that increased oxidative stress is a significant pathogenic mechanism of obesity-associated metabolic syndrome, the antioxidant potential of our samples has been investigated. Four tests were applied to establish the potential antioxidant capacity of olive mill wastewater polyphenol-enriched fractions (Table 3). The RO retentate showed the most promising activity in ABTS test (IC_50_ 6.9 ± 1.9 μg/mL). Interesting data were also found for NF retentate (IC_50_ value of 7.3 ± 1.6 μg/mL). NF permeate and RO permeate were the less active. The lipids peroxidation inhibitory activity was investigated by applying the β-carotene bleaching test. IC_50_ values of 25.1 ± 1.2 and 54.7 ± 3.3 μg/mL were found for RO retentate after 30 and 60 min of incubation, respectively. All samples exerted antioxidant effects in a concentration-dependent manner. In particular, in the DPPH test, the following trend of activity was found: RO retentate > NF retentate > MF permeate > feed > NF permeate > RO permeate. In the ABTS test, the same trend was observed.

## 4. Discussion

In recent years, increasing attention has been devoted to the possibility of valorising the olive oil extraction residues. In this contest, the use of pressure-driven membrane processes is considered as promising for the recovery, purification and concentration of polyphenols from OMWs [42].

In the present study, a combination of MF, NF and RO membranes operating in selected operating conditions was investigated for the selective separation and recovery of phenolic compounds from OMWs. The productivity of the MF membrane (steady-state permeate flux higher than 20 kg/m^2^⋅h) was of the same order as acetate cellulose membranes with a pore size of 0.2 μm [43]. Russo [25] reported higher productivities (of about 50 L/m^2^⋅h) for ceramic membranes with pore size of 0.45 and 0.8 μm. The productivity of the NF membrane, with a steady-state flux of about 1 kg/m^2^⋅h, can be compared with that of the NF90 membrane, an aromatic polyamide membrane with a MWCO of 200 Da [26]. The permeate fluxes measured in RO were much lower than those reported by Russo [25] in the concentration of UF permeates with composite polyamide membranes (20–25 L/m^2^⋅h).

By referring to the separation of phenolic compounds, UHPLC data (Table 1) show that all compounds are highly recovered in the MF permeate. For the NF membrane, the measured rejections (Figure 5) are strongly correlated with the molecular weight of phenolic compounds. In particular, the retention towards tyrosol was 4.8%, while phenolic compounds with molecular weight higher than 194 g/mol were completely retained. As expected, all compounds were completely or almost completely rejected by the RO membrane. Although the sieving mechanism is mainly involved in the separation of phenolic compounds with NF and RO membranes, in practice, the MWCO of the membrane cannot be considered an absolute barrier. Indeed, the solubility of the solutes and the hydrophobicity of the membrane surface are key factors which affect the separation mechanism, reducing the impact of MWCO [44]. Therefore, the measured rejections cannot be explained on the basis of steric considerations alone. The nature of the molecules is also crucial for such recovery efficiency. Phenolic-based compounds present aromatic rings and aliphatic chains that produce a hydrophobic profile. This leads to the attraction of water molecules, an increased volume of the molecules and the restriction of permeation through membrane pores due to the “polarity resistance” phenomenon [44]. In addition, phenolic compounds may interact together and with retained macromolecules to form large sized particles which can be adsorbed on the membrane surface, increasing the resistance to permeation [45].

The concentration of phenolic compounds in the RO retentate was about 3 g/L, with hydroxytyrosol (1522.2 ± 7.3 mg/L), tyrosol (519.0 ± 6.2 mg/L) and oleuropein (510.0 ± 5.5 mg/L) the most abundant compounds. These values were much higher than those reported in the final NF retentate (86 mg/L) of the integrated process developed by Cassano et al. [27] and in the RO retentate (225 mg/L) of the combined UF-NF-RO process developed recently by Sygouni et al. [46].

The concentration of phenolic compounds in the RO permeate was about 26 mg/L, showing that the polyphenols were almost completely recovered in the concentrate streams. In agreement with data reported by Sygouni et al. [46], the final stream can be reused for irrigation purposes.

Worldwide, the prevalence of obesity has notably increased due to lifestyle and to the increased consumption of foods rich in carbohydrates and fats [47]. Obesity, especially visceral adiposity, is an underlying risk factor for cardiovascular disease, which increases the incidence of various diseases, including diabetes, atherogenic dyslipidaemia, insulin resistance, hypertension, atherosclerosis, hypercholesterolemia and hyperglycaemia [48].

Several studies have revealed that obesity is associated with increased metabolic risk and altered redox state [49]. Oxidative stress can be a consequence but also a trigger of obesity. Chronic hyper-nutrition with a high intake of carbohydrates and lipids stimulates intracellular pathways, leading to oxidative stress through multiple mechanisms, including protein kinase C activation, superoxide generation from NADPH oxidases, glyceraldehyde autoxidation, oxidative phosphorylation, and hexosamine and polyol pathways [50,51,52]. Other factors that contribute to oxidative stress in obesity are abnormal post-prandial ROS generation, low antioxidant defences hyperleptinemia, chronic inflammation, and tissue dysfunction. Strategies to lower oxidative stress in obesity include weight loss, physical activity, and a supplementation with antioxidants. The plant kingdom is a rich source in which to search for new natural and effective hypoglycaemic and anti-obesity compounds. Furthermore, having compounds that are also excellent antioxidants would allow us to have multifunctional agents useful for the prevention and/or treatment of this disease.

The chemical analyses of olive mill wastewater polyphenol-enriched fractions obtained in this study revealed that, except for 4-hydroxyphenyl acetate, RO retentate is the fraction characterized by the highest content of bioactive compounds. In particular, the content of hydroxytyrosol in this fraction was about five times higher than its content in the feed. Other abundant compounds in RO retentate are tyrosol, oleuropein, verbascoside, vanillic acid, and luteolin.

Numerous studies have highlighted that hydroxytyrosol is able to protect against oxidative damage, to inhibit the inducible form of nitric oxide synthase, NADPH oxidase, 5-lipoxygenase, and cyclooxygenase. Moreover, hydroxytyrosol modulated the release of tumour necrosis factor-α and other pro-inflammatory mediators [53,54,55,56].

Hadrich et al. [57] studied the inhibitory effects of hydroxytyrosol and oleuropein against α-amylase and α-glucosidase, which are key enzymes related to carbohydrates digestion and elevation of blood glucose levels. The inhibition of these enzymes might be recognized as a therapeutic approach to reduce hyperglycaemia. In fact, the control of post-prandial hyperglycaemia is critical in the prevention and early intervention of type 2 diabetes management. In this work, hydroxytyrosol showed the strongest α-glucosidase inhibitory activity with an IC_50_ value of 150 μM and a mild α-amylase inhibition. This value is higher than of the positive control acarbose (IC_50_ value of 200 μM) and that of oleuropein (IC_50_ value of 400 μM). In a recent review article, Vlavcheski et al. [58] summarized in vitro and in vivo studies that have investigated the antidiabetic activity of hydroxytyrosol. This compound showed insulin-like effects on insulin target cells, including hepatocytes, adipocytes, and muscle cells, protective effects against oxidative stress, hyperlipidaemia, and hyperglycaemia in vivo models. Luteolin is one of the flavones rich in many plants that has been proven to possess strong antioxidant and anti-inflammatory effects as well as to improve insulin resistance in diet-induced obese mice [59]. Moreover, luteolin enhanced blood glucose, hemoglobin A1c, insulin, and homeostasis model assessment of insulin resistance levels. Verbascoside, isolated from the roots of *Clerodendrum bungei*, exhibited stronger α-glucosidase inhibitory effects (IC_50_ value of 0.5 mmol/L) than acarbose (IC_50_ value of 14.4 mmol/L) [60]. Wu et al. [14] demonstrated the anti-obesity properties of verbascoside by exploring its lipase inhibitory activity. The polyphenolic compound was shown to be a non-competitive lipase inhibitor.

Because the structure of verbascoside contains hydroxyl and galloyl groups, the binding of this compound to lipase may be due to hydrophobic interactions with the interior hydrophobic groups of the enzyme as well as to the formation of hydrogen bonds between the hydroxyl groups of verbascoside and the polar groups of lipase.

The physico-chemical characteristics of the RO permeate suggest its potential reuse for agriculture irrigation purpose as a valid alternative to fresh-water irrigation, especially in arid and semi-arid regions [61].

## 5. Conclusions

The combination of membrane-based operations, including MF, NF and RO, has been investigated in order to develop a sustainable process for the recovery of phenolic compounds and reduction in the environmental pollution caused by olive mill wastewaters.

The proposed process allowed us to produce a concentrated fraction (RO retentate) containing 3 g/L of phenolic compounds, with hydroxytyrosol, tyrosol, and oleuropein, the most abundant compounds. This fraction showed the best antioxidant activity in the ABTS test as well as the best hypoglycaemic activity. In conclusion, our data suggest that olive mill wastewater polyphenol-enriched fractions obtained by membrane processes are effective in scavenging radicals and protecting lipid-oxidations and in inhibiting key enzymes such as lipase, α-amylase, and α-glucosidase, useful therapeutic targets for the development of functional products for obesity and diabetes type 2 prevention.

## Figures and Tables

**Table 1:** Analysis of phenolic compounds (mg/L) in MF, NF and RO samples by UHPLC.

Phenolic Compounds	Feed	MF-P	NF-R	NF-P	RO-R	RO-P
Caffeic acid	8.1 ± 0.5 ^c^	7.6 ± 0.5 ^d^	27.7 ± 1.4 ^b^	1.8 ± 0.2 ^e^	45.7 ± 1.2 ^a^	0.5 ± 0.03 ^f^
*p*-Coumaric acid	6.4 ± 0.4 ^c^	4.3 ± 0.2 ^d^	12.2 ± 0.8 ^b^	2.6 ± 0.2 ^e^	35.9 ± 1.6 ^a^	nd
Ferulic acid	6.9 ± 0.6 ^c^	6.3 ± 0.3 ^d^	20.1 ± 1.1 ^b^	nd	51.3 ± 1.4 ^a^	nd
Luteolin	15.2 ± 0.6 ^c^	13.7 ± 1.1 ^d^	71.5 ± 2.7 ^b^	nd	82.8 ± 3.1 ^a^	nd
4-Hydroxyphenyl acetate	72.6 ± 1.8 ^a^	67.0 ± 2.6 ^b^	29.6 ± 1.2 ^e^	64.0 ± 3.2 ^c^	57.1 ± 1.2 ^d^	nd
Hydroxytyrosol	373.3 ± 4.8 ^c^	320.1 ± 5.8 ^d^	1017.5 ± 8.8 ^b^	268.3 ± 1.2 ^e^	1522.2 ± 7.3 ^a^	18.8 ± 1.2 ^f^
Oleuropein	106.8 ± 4.0 ^c^	85.2 ± 2.7 ^d^	263.2 ± 4.2 ^b^	nd	510.0 ± 5.5 ^a^	nd
Tyrosol	89.7 ± 2.1 ^c^	68.1 ± 5.1 ^d^	157.3 ± 4.3 ^b^	64.8 ± 1.2 ^e^	519.0 ± 6.2 ^a^	5.0 ± 0.6 ^f^
Vanillic acid	29.4 ± 0.3 ^c^	27.8 ± 1.7 ^d^	97.0 ± 2.5 ^b^	6.5 ± 0.9 ^e^	116.2 ± 3.1 ^a^	1.4 ± 0.1 ^f^
Verbascoside	26.7 ± 1.1 ^c^	18.0 ± 1.3 ^d^	82.8 ± 3.4 ^b^	nd	130.9 ± 1.2 ^a^	nd

MF, microfiltration; NF, nanofiltration; RO, reverse osmosis; P, permeate; R, retentate. Data are expressed as mean ± S.D.; Differences were evaluated by one-way analysis of variance (ANOVA) completed with a multicomparison Tukey’s test. Means in the same row with different small letters differ significantly (*p* < 0.05). nd: not detected.

**Table 2:** Hypoglycaemic and hypolipidemic activity [IC_50_ value (μg/mL)].

Sample	α-Amylase	α-Glucosidase	Lipase
Feed	287.9 ± 3.7 ^****^	167.0 ± 2.8 ^****^	245.7 ± 3.7 ^***^
MF-P	253.7 ± 3.6 ^****^	125.4 ± 2.7 ^****^	211.0 ± 3.4 ^***^
NF-R	122.6 ± 2.7 ^****^	116.0 ± 2.5 ^****^	183.1 ± 2.9 ^***^
NF-P	470.9 ± 4.7 ^****^	710.1 ± 5.7 ^****^	826.1 ± 6.0 ^***^
RO-R	65.3 ± 1.7 ^***^	66.2 ± 1.7 ^***^	175.6 ± 2.9 ^***^
RO-P	16.2%	26.8%	10.8%
Positive control			
Acarbose	50.1 ± 0.8	35.7 ± 1.3	
Orlistat			37.2 ± 1.2

MF, microfiltration; NF, nanofiltration; RO, reverse osmosis; P, permeate; R, retentate. Data are expressed as means ± S.D. (*n* = 3). Differences within and between groups were evaluated by one-way ANOVA followed by a multicomparison Dunnett’s test (α = 0.05): **** *p* < 0.0001, *** *p* < 0.001, compared with the positive control.

**Table 3:** In vitro antioxidant activity.

Sample	DPPH Test IC_50_ (μg/mL)	ABTS Test IC_50_ (μg/mL)	β-Carotene Bleaching Test IC_50_ (μg/mL)		FRAP Test (μM Fe (II)/g)
			*t* 30 min	*t* 60 min	
Feed	61.3 ± 3.5 ^****^	15.2 ± 1.5 ^***^	48.8%	34.0%	59.4 ± 1.7 ^**^
MF-P	53.5 ± 3.3 ^****^	7.7 ± 0.8 ^**^	50.0 ± 2.9 ^****^	40.1%	66.6 ± 1.8 ^*^
NF-R	52.6 ± 3.2 ^****^	7.3 ± 1.6 ^**^	32.3 ± 1.9 ^****^	69.0 ± 3.5 ^****^	86.5 ± 1.9
NF-P	146.9 ± 4.2 ^****^	112.8 ± 1.9 ^****^	95.4 ± 4.0 ^****^	31.2%	10.7 ± 1.7 ^****^
RO-R	38.0 ± 1.9 ^****^	6.9 ± 1.9 ^*^	25.1 ± 1.2 ^****^	54.7 ± 3.3 ^****^	91.9 ± 1.6
RO-P	310.2 ± 4.7 ^****^	17.5%	23.51%	19.2%	3.6%
Positive control					
Ascorbic acid	5.1 ± 0.9	1.7 ± 0.5			
Propyl gallate			0.09 ± 0.02	0.09 ± 0.01	
BHT					63.4 ± 2.5

MF, microfiltration; NF, nanofiltration; RO, reverse osmosis; P, permeate; R, retentate. Data are expressed as means ± S.D. (*n* = 3). Differences within and between groups were evaluated by one-way ANOVA followed by a multicomparison Dunnett’s test (α = 0.05): **** *p* < 0.0001, *** *p* < 0.001, ** *p* < 0.01, * *p* < 0.1 compared with the positive control.
